# Zhisou powder in treatment of postinfectious cough: A protocol for systematic review and meta-analysis

**DOI:** 10.1097/MD.0000000000023117

**Published:** 2020-11-20

**Authors:** Haiyang Cai, Weihong Li, Shixin Kang, Jing He, Peng Yu, Han Li

**Affiliations:** Basic Medical College, Chengdu University of TCM, Chengdu, Sichuan Province, China.

**Keywords:** effectiveness and safety, postinfectious cough, systematic review, zhisou powder

## Abstract

**Background:**

The pathogensis of postinfectious cough (PIC) is unkown, unsatisfactory clinical curative effects of conventional western medicine have been shown. Zhisou powder (ZP) is one of the most common prescriptions in traditional Chinese medicine for the treatment of PIC. However, the effects and safety also remain uncertain. We aim to systematically review the effectiveness and safety of ZP for PIC.

**Methods:**

We will search the following databases: PubMed, Embase, Cochrane Library, MEDLINE, the China National Knowledge Infrastructure , the Chinese Biomedical Literature Database, Cqvip Database, and Wanfang Data. The studies published from the inception of the database to May 2020 will be retrieved. The randomized controlled trials on ZP for PIC will be included. The primary outcomes were cough relief rate and cough resolution rate. We will perform the analyses using RevMan V.5.3 software.

**Results:**

This study will provide high-quality evidence of ZP for PIC in the effectiveness and safety.

**Conclusions:**

This systematic review will assess whether ZP is an effective and safe prescription for PIC.

## 1 Introduction

Postinfectious cough (PIC), a subacute cough lasting from 3 to 8 weeks, is a common disease in primary care affecting. approximately 40% of adults develop a PIC after an acute respiratory tract infection.^[1,2]^ On the cough-specific quality of life, PIC has a more severe effect than acute or chronic cough. Because PIC has not been recovered as quickly as acute cough and not been adapted like a chronic cough.^[3,4]^ The pathogenesis of PIC is considered to be multifactorial and has not been known.^[5]^ It is believed to be related to gastroesophageal reflux, airway inflammation, and epithelial disruption caused by Mycoplasmal pneumoniae, Bordetella pertussis, respiratory syncytial virus, rhinovirus, and influenza.^[1,2,6–8]^

Although PIC has affected the patient's quality of life. Unfortunately, there is no available guidance based on prospective, randomized, controlled trials in the treatment of PIC. Inhaled or oral corticosteroids, inhaled ipratropium, dextromethorphan, antihistamines, leukotriene-receptor antagonist montelukast, and central acting antitussive agents are being commonly used. But the result is unsatisfactory.^[2,9–11]^ To relieve PIC, a growing number of patients turn to traditional Chinese medicine (TCM).

ZP, a prescription created by the famous medical scientist Zhongling Cheng in the Qing Dynasty for prolonged cough caused by external evil invading the lungs, is composed of Jigeng (Platycodon grandiflorus), Ziwan (Aster tataricus L. f), Jingjie (Nepeta cataria L), Chenpi (Pericarpium Citri Reticulatae), Baibu (Stemona japonica (Bl.) Miq), and Baiqian(Cynanchum glaucescens (Decne.).^[12]^ The external evil including wet-evil, wind-evil, summer-damp-evil, cold-evil, dryness-evil, and fire-evil is similar to various pathogens, such as: bacteria, viruses, fungi, etc.^[13]^ The external evil invading the lungs refers to lung infections caused by these pathogens. Due to the remarkable curative effect, ZP is still widely used for exogenous cough.^[14,15]^

Currently, a growing number of randomized controlled trials (RCTs) find that ZP has a significant effect of PIC and obvious advantages over the western medicine group,^[16–20]^ however, the sample size of them is relatively small. As a result, it is difficult to get reliable conclusions. Therefore, this systematic review and meta-analysis aim to evaluate the clinical effects of ZP for PIC and to provide evidence-based medicine.

## 2 Methods

### 2.1 Study registration

“The protocol for this systematic review was registered on INPLASY (10.37766/inplasy2020.9.0096) and is available in full on the inplasy.com (https://doi.org/10.37766/inplasy2020.9.0096).” The review protocol will be strictly enforced according to the Preferred Reporting Items for Systematic Reviews and Meta-Analyses Statement (PRISMA-P).

### 2.2 Ethics and dissemination

All data for this systematic review protocol have been published online and therefore the ethical approval is not needed.

### 2.3 Inclusion criteria

#### 2.3.1 Types of studies

All RCTs about ZP for PIC will be included regardless of language. The following studies: case series, quasi-RCTs Case reports, non-RCTs, cell experiments, animal experiments will be excluded.

#### 2.3.2 Participants

Participants who have been diagnosed with PIC will be included and regardless of gender, age, ethnicity, economic status, or restrictions, educational.

#### 2.3.3 T*ypes of interventions*

The experimental group only used ZP or combined with interventions of the control group. The control group used placebo control or no

Treatment or conventional medication, such as corticosteroids, ipratropium, dextromethorphan, antihistamines.

#### 2.3.4 Types of outcome measures

The primary outcomes were cough relief rate and cough resolution rate. Secondary outcomes include cough resolution time, cough relief time, change from baseline in TCM symptom score.

### 2.4 Exclusion criteria

The following literature will be excluded: the studies that complete data can not be obtained; the studies that data is wrong; the studies with incorrect intervention methods or random methods, etc. For duplicate literature, we will only pick 1 of them.

### 2.5 Search strategy and study selection

#### 2.5.1 Search strategy

The following electronic databases will be comprehensively searched including: PubMed, Cochrane Library, EMBASE, MEDLINE, China National Knowledge Infrastructure, Chinese Biomedical Literature Database, Cqvip Database, and Wanfang Data. All the literature retrieved is from the time when the database establishment to 20 May 2020. There are no language restrictions or regional restrictions. The search terms include “postinfectious cough”, “post-infectious cough”, “post-viral cough”, “cough post influenza”, “postcold cough”, “Zhisou Powder”, “Chinese medicine”, “TCM”, and “randomized clinical trials”.

#### 2.5.2 Study selection

**Figure 1:**
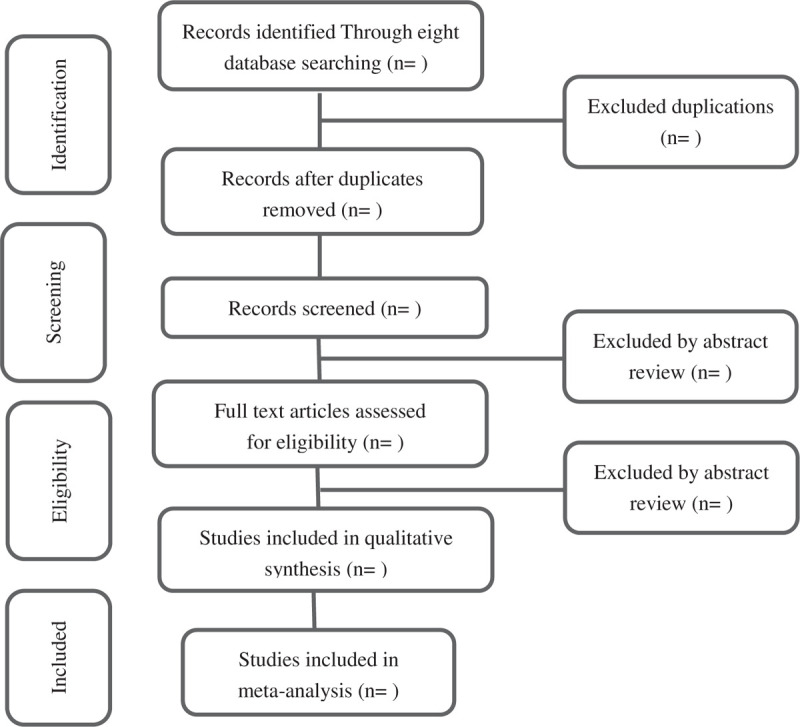
Flow diagram of the study selection process.

Two researchers independently search and use EndNote X7 software for management. The researchers will eliminate duplicate or unrelated literature by reading the title and abstract, and then confirm eligible Studies by reviewing the full text. If any dispute occurs, the divergence will be resolved by consulting the third researcher. The missing information will be supplemented by contacting the original author. The process of study selection is strictly performed according to the PRISMA flow diagram (Fig. 1).^[21]^

### 2.6 Data extraction

Data extraction is fulfilled by 2 researchers independently. Data extracted include author, gender, age, publication date, country, sample size, intervention details, follow-up information, safety, outcomes, and so on. Any disputes about data extraction will be resolved through consensus.

### 2.7 Assessment of risk of bias

The Cochrane Collaboration's tool will be used to assess the risk of literature bias.^[22]^ The methodological quality will be assessed by 2 investigators independently using RevMan 5.3.0. The following 7 aspects will be evaluated. Including: random, blinding of participants and investigators, sequence generation, allocation concealment, the blindness of outcome assessments, selective outcome reporting, incomplete outcome data, and other biases. As a result, every included study will be assessed as low, unclear, or high bias.

### 2.8 Statistical analysis

For data analysis, RevMan 5.3.0 that is provided by the Cochrane Collaboration will be used. We will use the Chi-squared test and I^2^ statistic to evaluate the heterogeneity of similar studies. If *P* ≥ .05 and I^2^≤ 50%, we believe it is low heterogeneity. As result, we will use a fixed-effects model. If *P* < .05 and I^2^ > 50%, it means there is heterogeneity. We will use a random-effects model. For the enumeration data, odds ratio with a 95% confidence interval will be used to represent. We will use mean difference with 95% confidence interval to express the measurement data. The statistical significant difference is thought of as *P* < .05.

If the studies show significant heterogeneity. Subgroup analysis will be performed to explore the source of heterogeneity. Furthermore if necessary, a sensitivity analysis will be performed.

### 2.9 Publication bias

If more than 10 studies are finally included in the meta-analysis. We will assess whether there is a reporting bias using a funnel plot.

## 3 Discussion

PIC is a disease with a high incidence, which seriously affects the quality of life of patients.^[1]^ However, the effects of conventional medication such as: corticosteroids, ipratropium, dextromethorphan, antihistamines are not satisfactory.

TCM believes that PIC is caused by external evil invading the lungs and has extensive experience in treating PIC. Clinical studies have found that ZP has a significant effect on PIC.^[18,19]^

However, there is no evidence-based medical evidence to prove the safety and efficacy of ZP in treating PIC. Therefore, the purpose of this study is to provide high-quality evidence on the efficacy and safety of ZP in treating PIC.

## Author contributions

**Conceptualization:** Haiyang Cai, Weihong Li, Shixin Kang.

**Data curation:** Peng Yu.

**Funding acquisition:** Weihong Li.

**Investigation:** Shixin Kang.

**Methodology:** Haiyang Cai, Shixin Kang.

**Project administration:** Haiyang Cai, Weihong Li.

**Software:** Jing He.

**Supervision:** Han Li.

**Validation:** Weihong Li.

**Writing – original draft:** Haiyang Cai, Shixin Kang.

**Writing – review & editing:** Haiyang Cai, Shixin Kang.
